# Management of unilateral temporomandibular joint ankylosis & orthomorphic correction in a patient with Marfan syndrome: A rare case report

**DOI:** 10.1016/j.ijscr.2020.09.033

**Published:** 2020-09-11

**Authors:** B.M. Rudagi, Jain Rishabh, Merchant Arif, Chourasia Namrata, Naikwade Shahbaaz, Bhavar Gaurav

**Affiliations:** a Professor and Head of Department, Department of Oral and Maxillofacial Surgery, JMF’s ACPM Dental College and Hospital, Dhule, India; b Post-Graduate Student, Department of Oral and Maxillofacial Surgery, JMF’s ACPM Dental College and Hospital, Dhule, India

**Keywords:** Marfan syndrome, Orthomorphic, Ankylosis, Obstructive sleep apnea, Temporomandibular joint, Case report

## Abstract

**Introduction:**

Marfan syndrome (MFS) is a disorder of the connective tissue that is inherited in an autosomal dominant fashion and is caused by mutations in the gene coding for fibrillin-1 (FBN1). This condition commonly affects the skeletal system, pulmonary system, ocular system and the cardiovascular system. Surgical intervention is often needed to correct the various deformities affecting the patients to restore the function, improving the patient’s aesthetic appearance and quality of life.

**Presentation of case:**

It’s a rare case report of a young male patient suffering through unilateral temporomandibular joint ankylosis, obstructive sleep apnea and unaesthetic appearance while being a case of Marfan syndrome.

**Discussion:**

This paper highlights the various surgical procedures undertaken to correct the deformities affecting the individual and improving the overall health of the patient.

**Conclusion:**

Our patient’s management shows the necessity of a multidisciplinary, multi factorial and multi-faceted approach with early visual identification and diagnosis.

## Highlights

• Its a case report of a patient suffering from Unilateral TMJ Ankylosis, Marfan Syndrome & OSA.
• Surgical intervention is often needed to correct the various deformities affecting the patients to restore the function, improving the patient’s aesthetic appearance and quality of life.
• This paper highlights the various surgical procedures undertaken to correct the deformities affecting the individual and improving the overall health of the patient.
• Our patient’s management shows the necessity of a multidisciplinary, multi factorial and multi-faceted approach with early visual identification and diagnosis.

## 1 Introduction

Marfan syndrome (MFS) is a disorder of the connective tissue caused by mutations in the gene coding for fibrillin-1 (FBN1). It follows an autosomal dominant fashion of inheritance [1,2,4]. The incidence of Marfan’s Syndrome is estimated to be 2–3 per 10,000 individuals without any racial predilection [7].

It was described in 1896 by a French paediatrician, Antoine Bernard- Jean Marfan, in a 5-year-old girl named Gabrielle who presented with “spider’s legs” or dolichostenomelia. Marfan syndrome has been classified into two types, I and II using the Ghent diagnostic criteria [3].

The diagnosis is commonly considered if a young person presents with a tall, thin body type, long limbs, and funnel-shaped chest. This condition commonly affects the musculoskeletal system, pulmonary system, ocular system and the cardiovascular system [1]. Cardiovascular system complications include dilatation of the ascending aorta, mitral valve prolapse, which account for 95% of the deaths in patients. As for skeletal involvement, a tendency toward tall stature with long, slim limbs, pectus excavatum, muscle hypotonia, joint hypermobility, and scoliosis can be seen [9].

The predominance of obstructive sleep apnea (OSA) is considerably high. OSA may be a risk factor for aortic root dilatation. The mechanisms involved in the high prevalence of OSA in patients are not established. Increased upper airway collapsibility during sleep and high nasal airway resistance, due to maxillary constriction, the retruded mandible has been reported as possible causes [7].

Oral clinical findings consist of high arched palate with dental crowding, retrognathia and occlusal disturbance [1]. Joint hypermobility is a common finding in Marfan syndrome; however, only limited attention was paid in the past to temporomandibular joint (TMJ) dysfunction [6].

Surgical intervention is often needed to correct the various deformities affecting the patients to restore the function, improving the patient’s aesthetic appearance and quality of life [8]. This paper highlights the various surgical procedures undertaken to correct the deformities affecting the individual and improving the overall health of the patient. The work has been reported in line with the SCARE criteria [11].

## 2 Clinical presentation

A 27-year-old male patient reported to the Department of Oral & Maxillofacial Surgery with a chief complaint of reduced mouth opening.

Patient complained of restricted mouth opening of approximately 5 mm and had difficulty in speech and chewing. On examination, the patient revealed history of trauma to the left side of the face in childhood for which patient received no treatment.

**Fig. 1:**
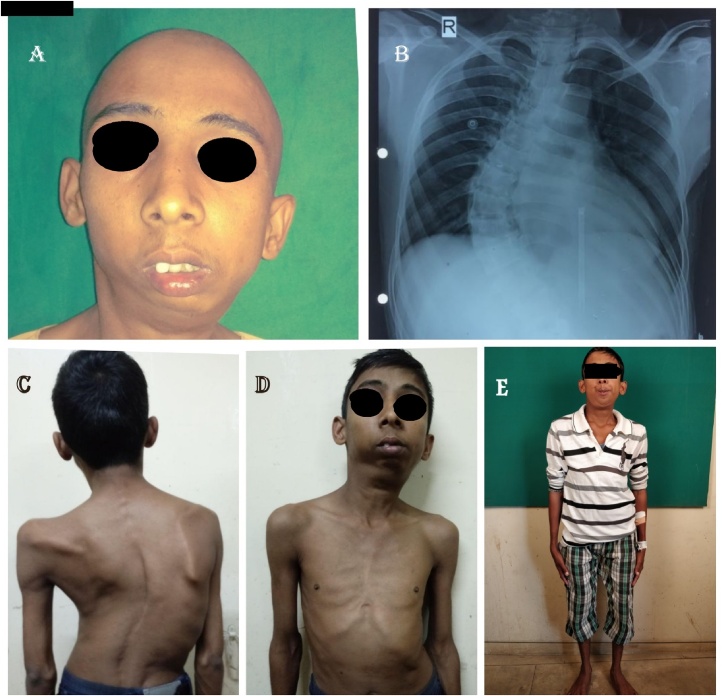
Pre-operative Photographs. A: Frontal Profile, B: Chest Radiograph, C,D: Skeletal Features, E: Full Body Photograph.

**Fig. 2:**
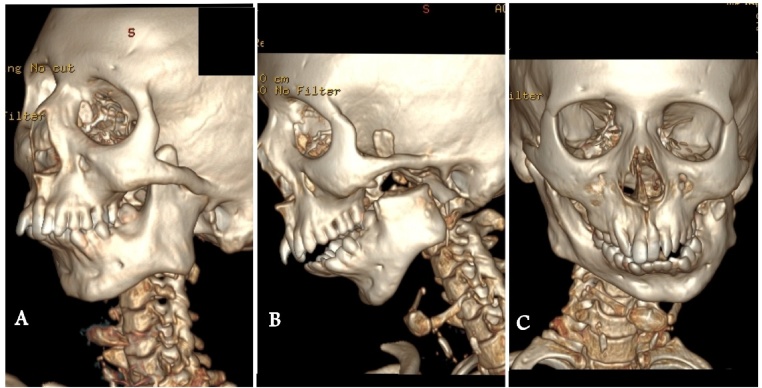
Computed Tomographic Images. A: Pre – op TMJ Ankylosis. B: Post – op TMJ Ankylosis. C: Pre – op Orthomorphic correction.

Head and neck examination revealed a convex profile with a typical bird face appearance. Intraorally, high arched palate, anterior crowding in both the arches and anterior open bite was present. The patient had an increased overjet. Also evident were mandibular retrognathia, and fullness of cheek on the left side, loss of mandibular curvature on the right side. Facial asymmetry was seen due to deviation of the chin to the left side (Fig. 1). On clinical and radiographic examination, unilateral temporomandibular joint ankylosis was diagnosed on the left side (Fig. 2).

The patient also gave history of difficulty in breathing while sleeping and irregular sleep cycles, the patient was assessed using the Berlin questionnaire for obstructive sleep apnea, where he was designated at high risk.

On general examination, the patient was of short stature, being 4 feet 5 in. in height, weighing 38 kgs. Built of the patient was ectomorphic with long upper and lower extremities (Genu varum). The patient had disproportionately long arms and legs as compared with the trunk, and his arm span was more than his height by about 2 in. with an increased floor to pubis measurement. The chest was pigeon-like with prominent ribs. Scoliosis and kyphosis were also noticed (Fig. 1). Suspecting a syndromic condition, the patient was further evaluated.

A special clinical test for the evaluation of hyperextensibility included thumb (Steinberg) sign and wrist (walker sign) which were both positive.

On overall assessment, the patient was diagnosed as a variant of Marfan syndrome using the 2010 revised Ghent nosology [4]. Both the clinical and radiographic findings were suggestive of Marfan syndrome. The patient was then referred for ophthalmologic and cardiac evaluation, which confirmed the diagnosis.

According to the Ghent nosology, five major and four minor skeletal system criteria, one major and one minor cardiovascular system criteria and one major and one minor dural criterion were found in this case [4].

The patient was finally diagnosed with left temporomandibular joint ankylosis, obstructive sleep apnea and Marfan syndrome. While temporomandibular joint ankylosis and Marfan syndrome were special conditions not related by each other, obstructive sleep apnea was caused due to several contributing factors from both the diseases. Restricted condylar growth and reduced mouth opening due to ankylosis along with high palatal arch and retrognathic mandible being features of Marfan syndrome contributed towards the prevalence of obstructive sleep apnea in this case.

**Fig. 3:**
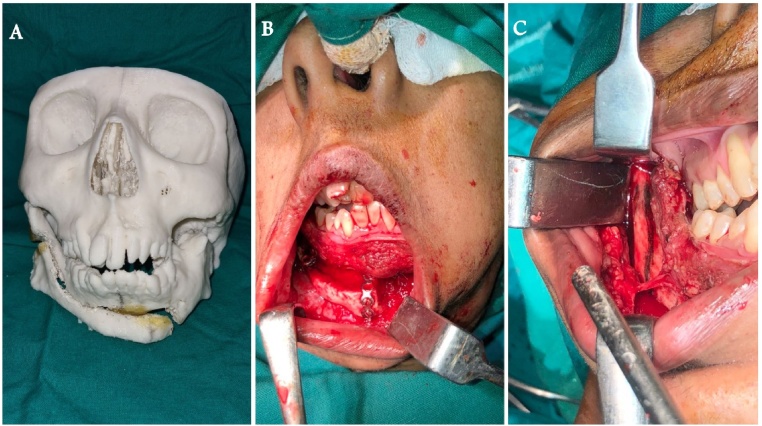
Intra-Operative Images. A: STL Model pre-orthomorphic surgery. B: Intra Op Orthomorphic Surgery. C: Intra Op Orthomorphic Surgery.

**Fig. 4:**
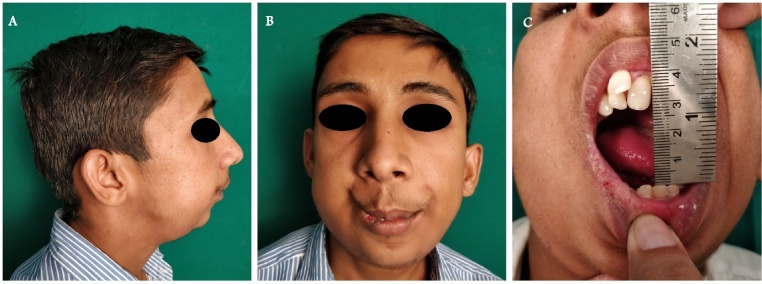
Post-Operative Images. A: Lateral View. B: Frontal View. C: Mouth Opening photograph.

Following complete examination and assessment, the patient was initially prepared for release of left temporomandibular joint ankylosis where interpositional gap arthroplasty with temporalis Myofascial flap was performed. Due to ankylosis the normal mandibular curvature was lost, and the corpus became straightened, the gonion shifted medially towards the side of deficiency, and the mandibular angle was obtuse, resulting in mandibular dysmorphology. Orthomorphic surgery was then performed to correct the facial deformity and also to correct obstructive sleep apnea which included advancement and sliding genioplasty (Fig. 3). Long term post-operative follow up for up to 3 years at every six months interval using berlin questionnaire ensured reduction of obstructive sleep apnea from high risk to a low-risk case (Fig. 4). We found out that OSA symptoms like snoring, stoppage of breathing while sleeping, difficulty in sleeping had reduced significantly.

## 3 Discussion

Marfan syndrome is a condition that has not only systemic manifestations but also affects the appearance and quality of life of an individual.

The recently revised criteria, known as the *Ghent criteria*, are based on a combination of major and minor clinical manifestations. According to the Ghent nosology, five major and four minor skeletal system criteria, one major and one minor cardiovascular system criteria and one major and one minor dural criterion were found in this case [4].

In the case described herein, there was no known family history of the syndrome. However, the patient exhibited features involving three different organ systems, thereby meeting the Ghent criteria for a diagnosis of Marfan syndrome.

Constriction of the maxilla, retrognathia and crowded dentition represent those oral manifestations for which patients seek aesthetic correction. Such cases tend to show high-arched palate accompanying narrow nasal airway and compensatory mouth breathing which alters their natural head position. High nasal airway resistance also increases the chances of obstructive sleep apnoea [5].

Maxillary constriction, irrespective of its severity, might play a role in the pathophysiology of OSA for the strong relationship with low tongue posture that could lead to oropharynx airway narrowing. In addition, increased upper airway collapsibility during sleep related to the connective tissue defect typical of the syndrome play a decisive role in the OSA pathogenesis [7].

In a growing patient with unilateral temporomandibular joint ankylosis associated with facial asymmetry, the mandible on the affected side is short, the contralateral side is long and flat, and the chin is deviated to the ankylosed side and is also retrognathic [10]. During the release of ankylosis, i.e. resection of the ankylosed condyle with the coronoid process, a gap may be created, and this can cause improvements in the mandibular movements [10].

The patient underwent surgery for orthomorphic correction of the mandibular asymmetry following the release of the ankylotic mass. The main advantage of this orthomorphic correction is its ability to alter the entire area of contour defect in any desired dimension as a single unit. A significant limitation of this technique was the inability to establish perfect symmetry. This was because it repositioned the deformed mandibular segment but did not correct the straightened contour of the body of the mandible. Modifications of this technique can be used in a number of ways, including as a technique for bridging small osseous defects of the mandibular body [2]. In our case, we advanced and did a sliding genioplasty to increase the pharyngeal lumen to aid breathing and relieve the patient of obstructive sleep apnea. No case has been recorded where orthomorphic correction was used to treat not only mandibular deformity but also obstructive sleep apnea.

A clinical case where multiple conditions independent of each other like Marfan syndrome and temporomandibular joint ankylosis in a patient together contributing towards the development of obstructive sleep apnea has been explained in this clinical paper and our experience at its effective management. Patients with such conditions often suffer through a lot of physical and esthetic issues. We were able to successfully help the patient with its most basic activities like speech, mastication, breathing and sleeping through surgical intervention and physiotherapy.

## 4 Conclusion

Our patient’s management shows the necessity of a multidisciplinary, multifactorial and multi-faceted approach with early visual identification and diagnosis.

A better understanding of the etiopathogenesis and adequate treatment techniques can lead to appropriate therapy decisions for the prevention or correction of such manifestations.

## Funding

None.

## Ethical approval

Ethical approval is exempted by the institution.

## Consent

“Written informed consent was obtained from the patient for publication of this case report and accompanying images. A copy of the written consent is available for review by the Editor-in-Chief of this journal on request”.

## Author contribution

**Table:** 

	Contributor 1	Contributor 2	Contributor 3	Contributor 4	Contributor 5	Contributor 6
Concepts	✓	✓	✓	✓	✓	✓
Design	✓	✓	✓			✓
Definition of Intellectual Content	✓	✓	✓	✓	✓	✓
Literature Search	✓	✓	✓	✓		
Clinical Study	✓	✓	✓		✓	✓
Experimental Study	✓	✓	✓		✓	
Data Acquisition	✓	✓	✓	✓		✓
Data Analysis	✓	✓	✓		✓	
Statistical Analysis	✓	✓	✓	✓	✓	✓
Manuscript preparation	✓	✓	✓	✓		✓
Manuscript editing	✓	✓	✓			
Manuscript review	✓	✓	✓	✓		✓
Guarantor	✓	✓	✓		✓	✓

## Registration of research studies

1. Not a clinical trial, it’s a case report.

## Guarantor

Dr. Rishabh H. Jain (https://orcid.org/0000-0002-7150-8819) Email Id: rishabh.j2003@yahoo.com JMF’s ACPM Dental College and Hospital, Dhule, Maharashtra, India 424001. Contact Number: 9870122101

## Provenance and peer review

Not commissioned, externally peer-reviewed.

## Declaration of Competing Interest

None.
